# Effective Management of Familial Erythrokeratoderma Using Topical Calcipotriol

**DOI:** 10.7759/cureus.87606

**Published:** 2025-07-09

**Authors:** Tamilmalar Kulandaivelavan, Manu Vidhya Harikumar, Leena Dennis Joseph, Adikrishnan Swaminathan

**Affiliations:** 1 Dermatology, Venereology and Leprosy, Sri Ramachandra Institute of Higher Education and Research, Chennai, IND; 2 General Pathology, Sri Ramachandra Institute of Higher Education and Research, Chennai, IND

**Keywords:** autosomal dominant, erythrokeratoderma, genodermatosis, keratinization disorder, progressive symmetrical erythrokeratoderma

## Abstract

Progressive symmetrical erythrokeratoderma (PSEK) is a rare genodermatosis that is inherited in an autosomal dominant pattern. It is clinically characterized by the insidious onset of well-defined, non-migratory, erythematous, hyperkeratotic plaques in a symmetrical distribution over the extremities. Hereby, we describe two cases of PSEK in a father and daughter, who presented with bilaterally symmetrical hyperpigmented scaly plaques, with skin biopsy findings suggestive of erythrokeratoderma. The skin lesions resolved with topical calcipotriol monotherapy, without the use of topical corticosteroids or oral medications. During telephonic follow-up, the patients reported that recurrences also responded well to topical calcipotriol, without the need for additional medication. This case report highlights the use of topical calcipotriol as a safe and effective therapeutic alternative for the treatment of erythrokeratoderma.

## Introduction

Erythrokeratodermas are rare genodermatoses that belong to the diverse group of keratinization disorders, characterized by erythematous, hyperkeratotic plaques on the skin [1]. They have an estimated prevalence of approximately one in 2,000,000 individuals [2]. They are categorized into two non-syndromic forms: erythrokeratoderma variabilis (EKV) and progressive symmetrical erythrokeratoderma (PSEK), and two syndromic forms: keratitis ichthyosis deafness syndrome and hystrix-like ichthyosis with deafness syndrome [3]. PSEK is an uncommon autosomal dominant disease with reduced penetrance and variable expressivity [4]. To date, fewer than 100 cases have been reported worldwide [5]. Here, we report two cases of PSEK within the same family.

## Case presentation

Case 1

**Figure 1:**
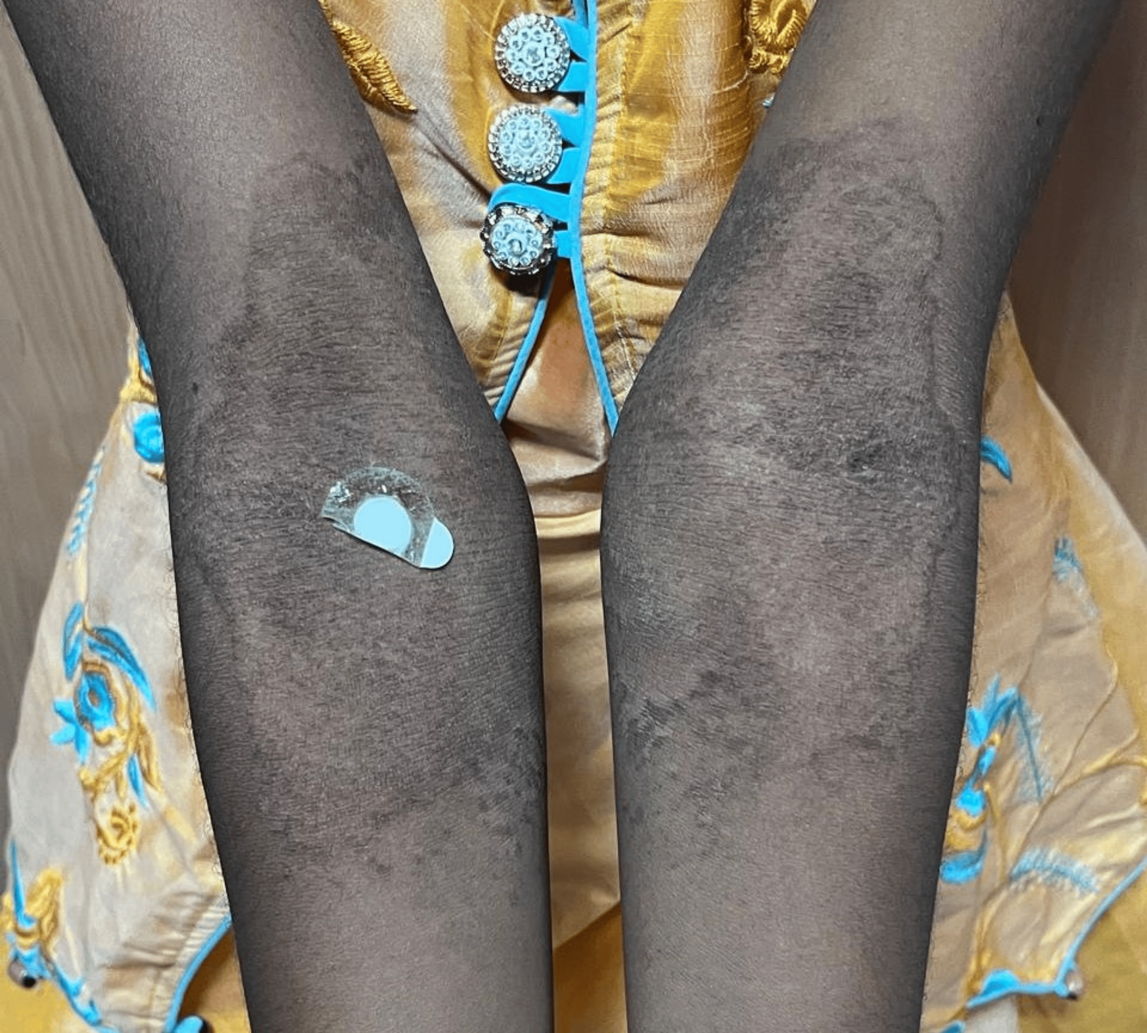
Well-defined hyperpigmented plaques over the bilateral cubital fossa

**Figure 2:**
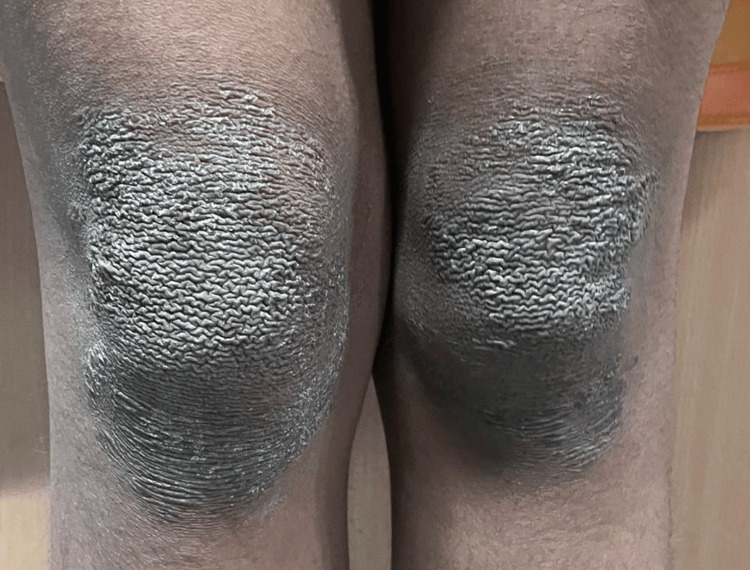
Hyperpigmented plaques with mild scaling over the bilateral knees

A 13-year-old female child, the firstborn of a non-consanguineous marriage, presented with persistent, asymptomatic, dark, raised skin lesions over both underarms, forearms, and knees, with peeling of skin over feet for five years. There was a history of similar complaints in her father. Examination revealed well-defined, hyperpigmented, mildly scaly plaques in symmetrical distribution over the bilateral axilla, cubital fossa, and knees (Figures 1-2).

**Figure 3:**
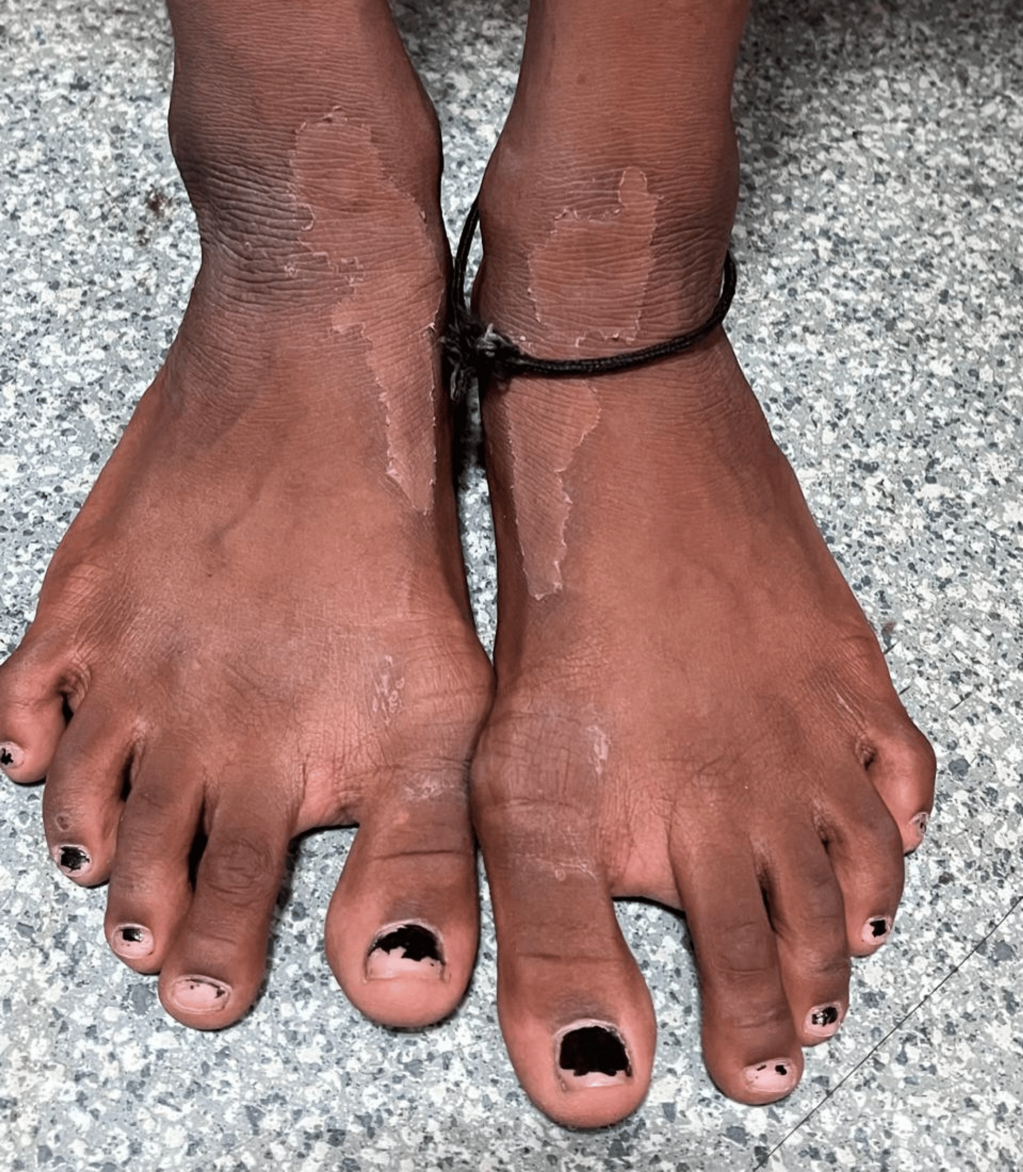
Exfoliation of the skin over the dorsum of the feet

Exfoliation of skin was noted over the face and dorsum of the feet (Figure 3).

**Figure 4:**
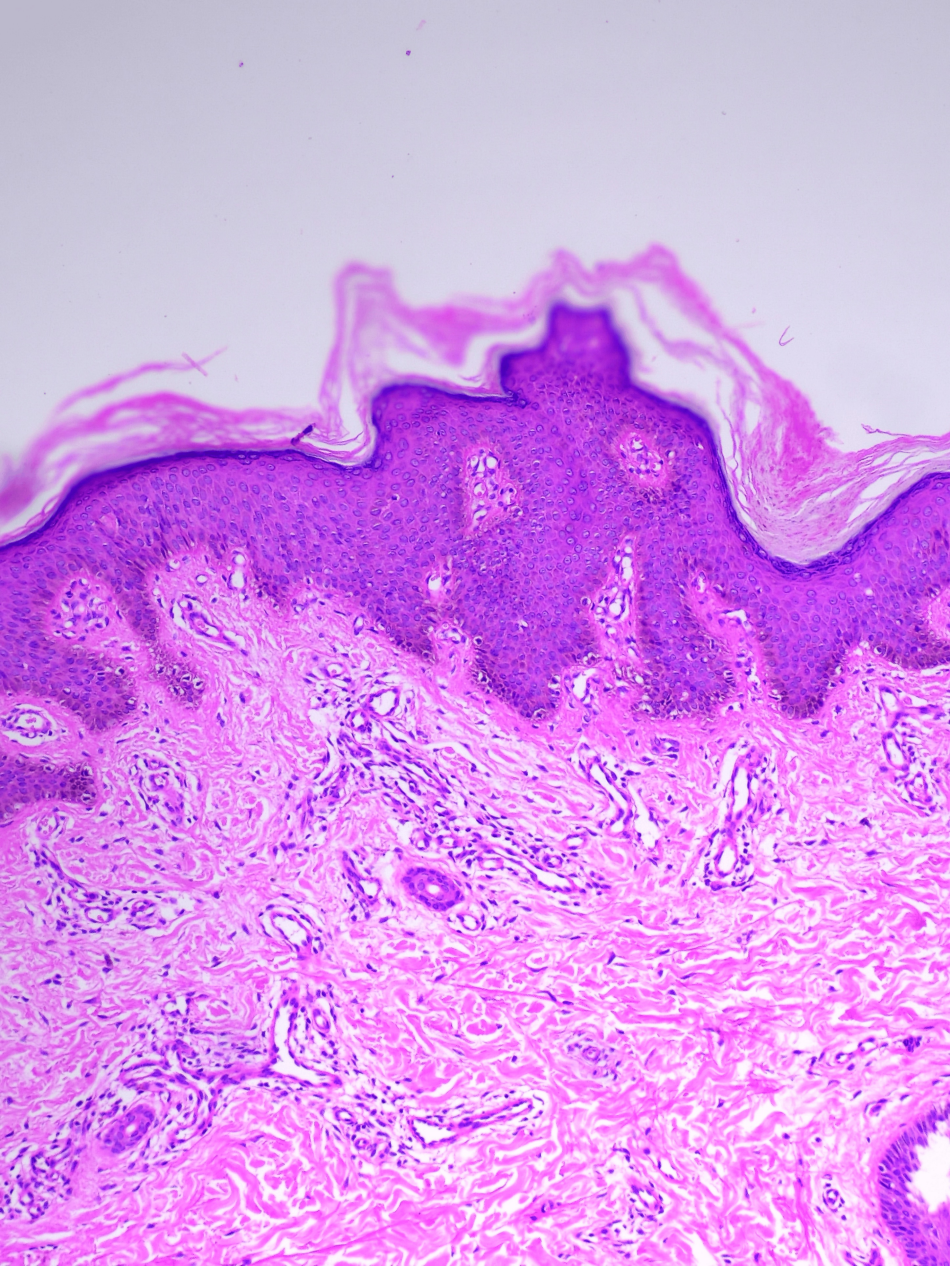
Histopathology image of the biopsy from the left knee Basket weave hyperkeratosis, focal parakeratosis, acanthosis and prominent papillomatosis (hematoxylin and eosin stain, 10x)

Scalp, oral cavity, palms, soles, nails, and genitalia were normal. Based on these clinical findings, a provisional diagnosis of PSEK was made, and a skin biopsy was performed, which revealed basket-weave hyperkeratosis, focal parakeratosis, acanthosis, and prominent papillomatosis, consistent with erythrokeratoderma (Figure 4).

She was started on moisturizer and topical calcipotriol and was planned for oral retinoids, but her skin lesions resolved in two months.

Case 2

**Figure 5:**
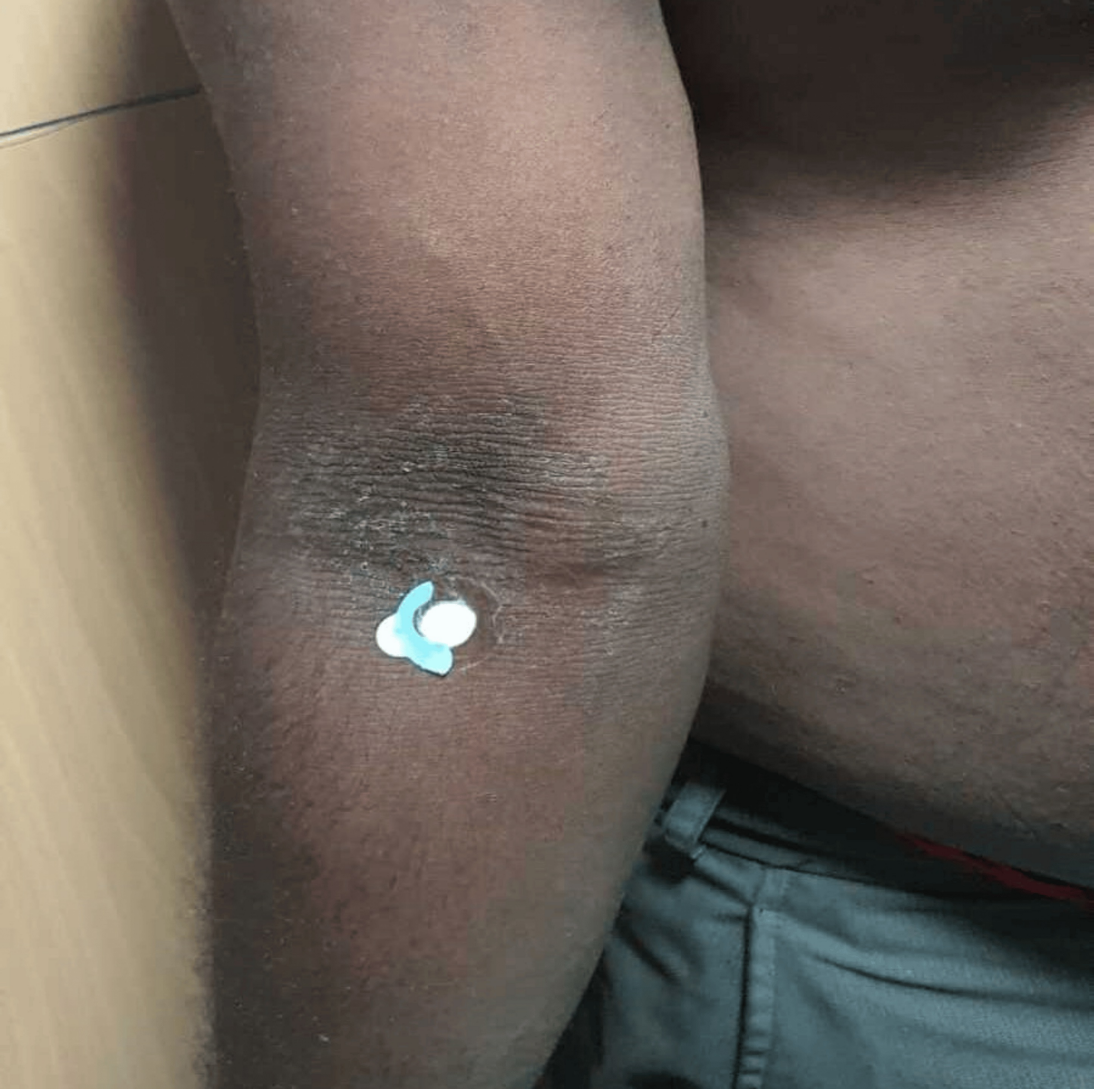
Hyperpigmented plaque with mild scaling over the right cubital fossa

**Figure 6:**
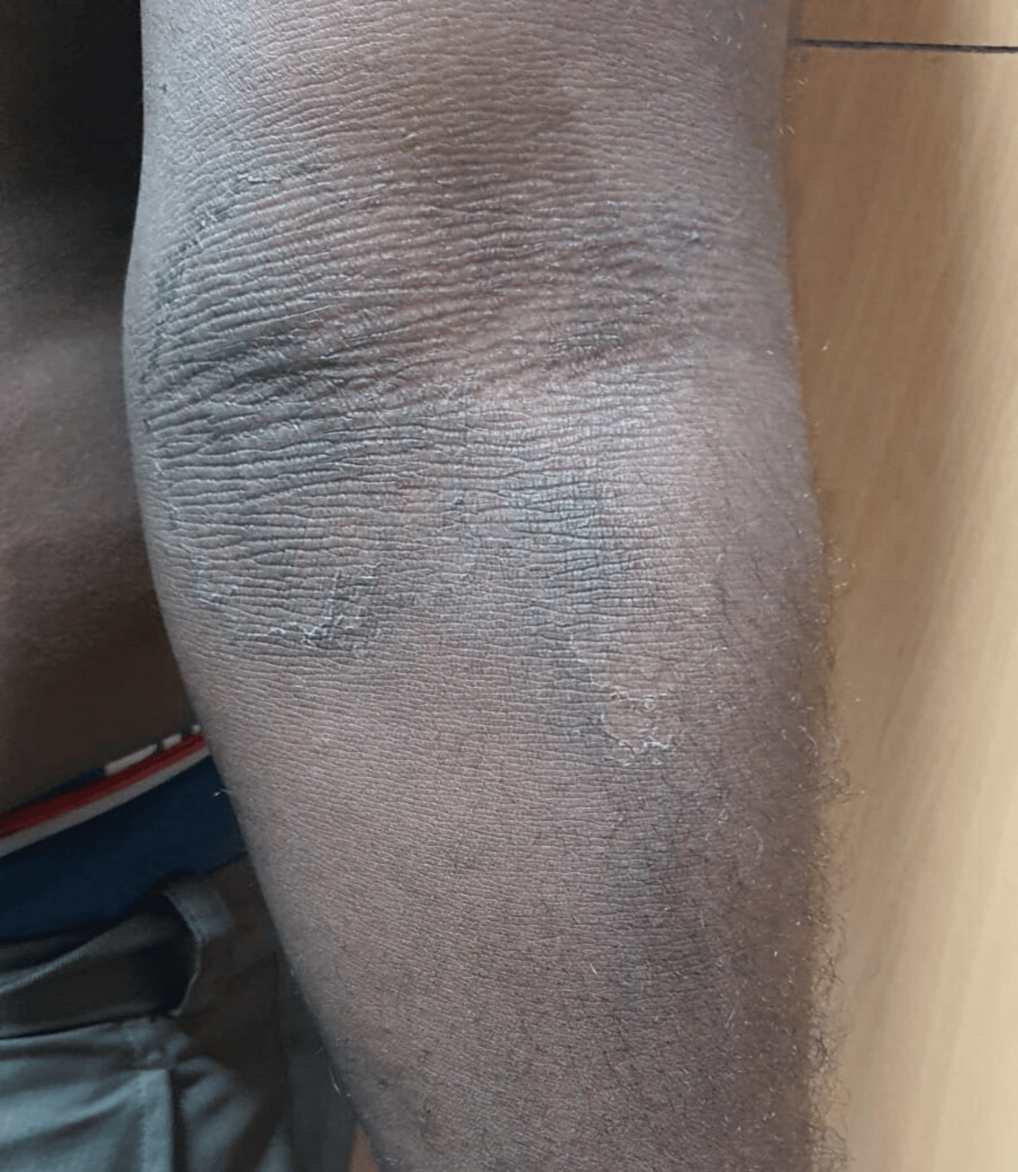
Well-defined hyperpigmented scaly plaque over the left cubital fossa

A 42-year-old male, father of the above-mentioned child, second-born of a non-consanguineous marriage, presented with similar lesions over the bilateral axilla and cubital fossa since childhood (Figures 5-6).

**Figure 7:**
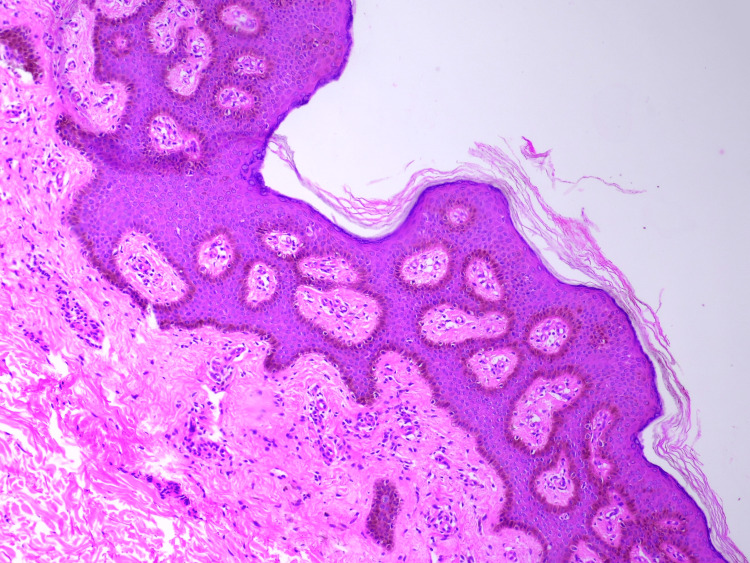
Histopathology image of the biopsy from the right arm Hyperkeratosis, acanthosis with mild papillomatosis (hematoxylin and eosin stain, 10x)

A skin biopsy revealed hyperkeratosis, acanthosis with mild papillomatosis, consistent with erythrokeratoderma (Figure 7).

He was also diagnosed with PSEK and was started on topical calcipotriol. Although oral retinoids were considered, he showed a good clinical response to topical calcipotriol alone.

## Discussion

PSEK, otherwise known as Gottron syndrome or Darier-Gottron syndrome, was initially described by Darier in 1911 and subsequently named by Gottron in 1923 [1]. It is generally an autosomal dominant genodermatosis caused by negative mutations in the GJB4 gene, which encodes connexin 30.3. A homozygous loss-of-function mutation of KRT83 has been encountered in its recessive form [6]. Studies have demonstrated that a mutation in the loricrin gene, located on chromosome 1q21.3, results in the accumulation of abnormal loricrin in the nucleus, thereby perturbing the normal process of keratinocyte apoptosis and contributing to the pathological thickening of the stratum corneum. Sporadic cases account for approximately 50 percent of the total diagnosed patients. In this case, the condition appears to follow an autosomal dominant pattern of inheritance. Research indicates that high mitotic activity is observed in the affected skin [1]. There is no gender predilection for this disease [4].

The skin is not affected at birth. The disease presents in infancy with large, fixed, erythematous, fine, scaly plaques in a symmetrical distribution, most commonly over the cheeks, shoulders, elbows, knees, dorsum of the hands and feet, and gluteal region, associated with mild pruritus [6,7]. Lesions increase in size and number with time and become stable after puberty. Palmoplantar keratoderma is observed in approximately 50 percent of patients [1]. Unlike EKV, which is characterized by migratory lesions that may be provoked by mechanical pressure and shows seasonal exacerbations, PSEK tends to present with symmetrical, fixed lesions and shows a higher prevalence of facial involvement and palmoplantar keratoderma [3,7]. Macfarlane et al. have described two sisters from the same family with erythrokeratoderma, where the younger sister had EKV and the elder one had PSEK, implying that they may be diverse presentations of a spectrum of disorders [8].

Existing literature shows an association of PSEK with high-arched palate, fissured tongue, pectus excavatum, symmetric syndactylism, keratosis pilaris, delayed intellectual milestones, ataxia, convulsions, narcolepsy, bilateral cortical cataract, deafness, peripheral neuropathy, and nephrotic syndrome [3,9]. Still, none of these features were observed in our patients. Autosomal recessive KDSR variants have been reported in association with collodion membrane, a harlequin ichthyosis-like presentation, and thrombocytopenia, as well as progressive myelofibrosis, thereby emphasizing the clinical variability and the importance of genetic evaluation [10]. Due to financial constraints, genetic analysis was not possible in our cases.

Traditional management of erythrokeratoderma includes the use of emollients, topical retinoids, corticosteroids, and keratolytics such as salicylic acid [1,6]. Oral retinoids, primarily acitretin at a dose of 0.5 to 1 mg/kg, are considered the treatment of choice. However, recurrence is common upon discontinuation, and potential side effects, particularly in pediatric patients, must be carefully considered. Phototherapy has also been tried [1]. A review of the existing literature revealed reports of significant improvement with topical calcipotriol, which acts by inhibiting keratinocyte proliferation and promoting differentiation [4,11]. Hence, it was tried in our patients. Although oral retinoids were initially planned, both patients demonstrated significant improvement with topical calcipotriol alone; therefore, oral retinoids were not initiated. These findings suggest that topical calcipotriol is an effective and safer alternative for managing hyperkeratotic lesions in erythrokeratoderma.

## Conclusions

PSEK is a chronic genodermatosis without a definitive cure. Enhancing clinical awareness is essential to prevent misdiagnosis, as early recognition can significantly improve the quality of life for patients and enable appropriate genetic counseling for affected family members. Considering the chronic and relapsing nature of the disease, topical calcipotriol may offer a safer, more cost-effective, and efficacious alternative to conventional therapies for the management of exacerbations.
